# A Bayesian many-facet Rasch model with Markov modeling for rater severity drift

**DOI:** 10.3758/s13428-022-01997-z

**Published:** 2022-10-25

**Authors:** Masaki Uto

**Affiliations:** The University of Electro-Communications, Tokyo, Japan

**Keywords:** Item response theory, Many-facet Rasch model, Rater effects, Rater drift, Bayesian modeling, Educational/psychological measurement

## Abstract

Fair performance assessment requires consideration of the effects of rater severity on scoring. The many-facet Rasch model (MFRM), an item response theory model that incorporates rater severity parameters, has been widely used for this purpose. Although a typical MFRM assumes that rater severity does not change during the rating process, in actuality rater severity is known to change over time, a phenomenon called *rater severity drift*. To investigate this drift, several extensions of the MFRM have been proposed that incorporate time-specific rater severity parameters. However, these previous models estimate the severity parameters under the assumption of temporal independence. This introduces inefficiency into the parameter estimation because severities between adjacent time points tend to have temporal dependency in practice. To resolve this problem, we propose a Bayesian extension of the MFRM that incorporates time dependency for the rater severity parameters, based on a Markov modeling approach. The proposed model can improve the estimation accuracy of the time-specific rater severity parameters, resulting in improved estimation accuracy for the other rater parameters and for model fitting. We demonstrate the effectiveness of the proposed model through simulation experiments and application to actual data.

## Introduction

In performance assessment, raters assess examinee outcomes or the processes for performing tasks. Such assessment is used in various fields and has attracted much attention as a means of measuring higher-order abilities, such as problem-solving, critical reasoning, and logical thinking skills (Linlin, 2019; Mislevy, 2018; Murtonen & Balloo, 2019; Palm, 2008; Shavelson, Zlatkin-Troitschanskaia, Beck, Schmidt, & Marino, 2019; Zlatkin-Troitschanskaia, Shavelson, Schmidt, & Beck, 2019). Performance assessments can be implemented in various formats, including essay writing, oral presentations, interview examinations, and group discussions.

A typical drawback of performance assessments is that the evaluation results depend on the severity (or leniency) of the raters, which decreases the reliability of the ability measurement (Deng, McCarthy, Piper, Baker, & Bolt, 2018; Eckes & Jin, 2021; Hua & Wind, 2019; Myford & Wolfe, 2003; Nguyen, Uto, Abe, & Ueno, 2015; Uto & Ueno, 2018). Therefore, the influence of rater severity needs to be considered in order to ensure reliable evaluation.

For this reason, item response theory (IRT) models that can estimate the abilities of examinees while considering the effects of rater severity have been proposed (Eckes & Jin, 2021; Jin & Wang, 2018; Linacre, 1989; Shin, Rabe-Hesketh, & Wilson, 2019; Uto & Ueno, 2018; Wilson & Hoskens, 2001). One such model is the many-facet Rasch model (MFRM) (Linacre, 1989). The MFRM and its extension models have been applied to various performance assessments to investigate rater effects, including rater severity, and to estimate examinee ability while removing the influence of those effects (Chan, Bax, & Weir, 2017; Deng et al., 2018; Hua & Wind, 2019; Jin & Wang, 2017; Kaliski et al., 2013; Linlin, 2019; Myford & Wolfe, 2004; Tavakol & Pinner, 2019).

These MFRMs generally assume that rater severity does not change during the rating process. However, it is known that this assumption is not often satisfied in practice, especially when each rater grades many examinees over a period of several hours or several days. The phenomenon in which rater severity changes over time is generally called *rater severity drift*, which is a component of rater drift, also called differential rater functioning over time (Casabianca & Lockwood, 2013; Harik et al., 2009; Hoskens & Wilson, 2001; Leckie & Baird, 2011; Myford & Wolfe, 2009; Park, 2011; Sgammato & Donoghue, 2017; Wilson & Case, 1997; Wind & Guo, 2019; Wind & Wesolowski, 2018; Wolfe et al., 2001; Wolfe, Myford, Engelhard, & Manalo, 2007). Several studies have proposed extension of MFRMs to investigate rater severity drift (Hoskens & Wilson, 2001; Myford & Wolfe, 2009; Wind & Wesolowski, 2018; Wolfe et al.,, 2001, 2007).

A simple extended model can be formulated as an MFRM that incorporates a time-specific parameter (Wind & Wesolowski, 2018; Wolfe et al.,, 2001; 2007), where the *time* indicates a time period for continuous rating, such as a rating session, an hour, or a day. This model enables investigation of severity changes averaged across raters. However, the severity drift of each individual rater cannot be assessed with this model due to the lack of information about the interaction between times and raters.

To resolve this problem, several MFRMs have been proposed that incorporate time-specific rater severity parameters (Myford & Wolfe, 2009; Wind & Wesolowski, 2018). These models provide each rater’s severity at each time point, enabling the severity drift to be determined for each rater. In these models, the time-specific rater severity parameters are estimated under the assumption of temporal independence. In practice, however, severities between adjacent time points are known to have temporal dependency. For example, several studies have reported that there are some raters whose severity remains stable over time, meaning that their time-specific severities are strongly correlated across time points (Casabianca & Lockwood, 2013; Hoskens & Wilson, 2001; Myford & Wolfe, 2009; Wilson & Case, 1997; Wind & Wesolowski, 2018). Furthermore, it is also known that the severity of some raters with severity drift tends to change gradually over time, meaning that their severity at a time point depends on that at the previous point and does not change randomly from point to point (Casabianca & Lockwood, 2013; Hoskens & Wilson, 2001; Wilson & Case, 1997). If rater severity is assumed to have this sort of time dependency, then we can expect that considering it will be helpful for improving the estimation accuracy of the time-specific severity parameters.

Therefore, we propose a Bayesian extension of the MFRM that assumes time dependency for the time-specific rater severity parameters, based on the approach of Markov modeling. In the proposed model, the time-specific severity parameters of each rater are modeled as a Markov chain, such that the severity at a time point depends on that at the previous point. Furthermore, we append rater-specific standard deviation parameters and a prior distribution on those parameters to the model. The rater-specific standard deviation parameters reflect the degree of the severity drift for each rater, and the prior distribution on those parameters reflects an analyst’s prior knowledge about how the extent of severity drift differs among raters. We adopt a Bayesian estimation method based on the No-U-Turn (NUT) Hamiltonian Monte Carlo (HMC), a popular Markov chain Monte Carlo (MCMC) algorithm (Hoffman & Gelman, 2014), as the parameter estimation method for the proposed model. The proposed model has the following features.

1. It can estimate time-specific rater severity parameters by considering their time dependency, resulting in more accurate estimation of the parameters than can be obtained by conventional models that assume their temporal independence.
2. It provides summarized information representing the degree of severity drift for each rater as the rater-specific standard deviation parameters.
3. It uses the prior distribution on the rater-specific standard deviation parameters to reflect our prior knowledge of how often rater severity drift occurs.
4. Because this model is a Bayesian extension of a conventional MFRM, its parameter estimates approach those of a non-Bayesian conventional MFRM when we have a large amount of data, which is a desirable property.
5. Improving the estimation accuracy of the time-specific rater severity parameters increases the estimation accuracy for other parameters and improves model fitting.

We demonstrate the effectiveness of the proposed model through simulation experiments and application to actual data.

## Many-facet Rasch models for rater severity drift

For scoring and analysis in various assessment settings, there has been an increase in the use of IRT (Lord, 1980). The Rasch model and the two-parameter logistic model are the most widely used IRT models, and they are applicable to test items for which responses are scored as correct or incorrect. Furthermore, there are various polytomous IRT models that are applicable to ordered categorical data, including the rating scale model (Andrich, 1978), the partial credit model (Masters, 1982), and the generalized partial credit model (Muraki, 1997). These types of traditional IRT models are applicable to two-way data consisting of *examinees* × *test items*. However, they cannot be applied directly to performance assessment data in which the examinees’ responses for test items are scored by multiple human raters. This is because we would then have three-way data consisting of *examinees* × *test items* × *raters*. Extended IRT models for such multi-faceted data have been proposed to address this problem (Eckes, 2015; Jin & Wang, 2018; Linacre, 1989; Shin et al., 2019; Uto & Ueno, 2018; Wilson & Hoskens, 2001). The MFRM is the most common type of model used for IRT with rater parameters (Linacre, 1989). Furthermore, there are various alternative models such as a two-parameter logistic model with rater severity parameters (Patz & Junker, 1999), generalized partial credit models incorporating various rater parameters (Uto, 2021b; Uto & Ueno, 2020), hierarchical rater models (DeCarlo, Kim, & Johnson, 2011; Patz, Junker, Johnson, & Mariano, 2002; Qiu, Chiu, Wang, & Chen, 2022), extensions based on signal detection models (DeCarlo, 2005; Soo Park & Xing, 2019), rater bundle models (Wilson & Hoskens, 2001), and trifactor models (Shin et al., 2019). However, this study focuses on the MFRM because it is the most widely used and well-established of these models.

Although conventional MFRMs assume that rater severity does not change during the rating process, this assumption is not satisfied when *rater severity drift* occurs as explained in “Introduction” section. Consequently, several studies have investigated extended MFRMs that are designed to detect rater severity drift (Hoskens & Wilson, 2001; Myford & Wolfe, 2009; Wind & Wesolowski, 2018; Wolfe et al.,, 2001, 2007).

A simple example of such an extension is the incorporation of a time facet parameter (Wind & Wesolowski, 2018; Wolfe et al., 2007). This model defines the probability that the performance of examinee *j* for item *i* will receive a score of *k* from rater *r* at time point *t* as

1 $$P_{ijrtk} = \frac{\exp {\sum}_{m=1}^{k}\left[D(\theta_{j} - \beta_{i} - \beta_{r} - \beta_{t} - d_{m}) \right]}{{\sum}_{l=1}^{K} \exp {\sum}_{m=1}^{l}\left[D(\theta_{j} - \beta_{i} - \beta_{r} - \beta_{t} - d_{m}) \right]},$$

where *𝜃*_*j*_ is the latent ability of examinee *j*, *β*_*i*_ is a difficulty parameter for item *i*, *β*_*r*_ is the severity of rater *r*, *β*_*t*_ is the parameter representing the averaged rater severity at time point *t*, and *d*_*m*_ is a step parameter denoting the difficulty of transitioning between scores *m* − 1 and *m*. *D* = 1.7 is the scaling constant used to minimize the difference between the normal and logistic distribution functions. This model enables investigation of the averaged changes in rater severity over time. However, because it ignores the interaction between time and raters, we cannot interpret the temporal changes of severity within each rater.

Several MFRMs incorporating time-specific rater severity parameters have been proposed to overcome this limitation. For example, Wind and Wesolowski (2018) has examined the following model:

2 $$P_{ijrtk} = \frac{\exp {\sum}_{m=1}^{k}\left[ D(\theta_{j} - \beta_{i} - \beta_{rt} - d_{m}) \right]}{{\sum}_{l=1}^{K} \exp {\sum}_{m=1}^{l}\left[D(\theta_{j} - \beta_{i} - \beta_{rt} - d_{m}) \right]}.$$

Here, *β*_*rt*_ is a time-specific severity parameter that represents the severity of rater *r* at time point *t*.

In addition, Hoskens and Wilson (2001) investigated the model

3 $$P_{ijrtk} = \frac{\exp {\sum}_{m=1}^{k}\left[ D(\theta_{j} - \beta_{i} - \beta_{irt} - d_{im}) \right]}{{\sum}_{l=1}^{K} \exp {\sum}_{m=1}^{l}\left[D(\theta_{j} - \beta_{i} - \beta_{irt} - d_{im}) \right]},$$

in which *β*_*irt*_ gives the time-specific rater severity parameter for each item, representing the severity of rater *r* for item *i* at time point *t*, and *d*_*im*_ is an item-specific step parameter denoting the difficulty of transitioning from score *m* − 1 to *m* for item *i*.

These models provide each rater’s severity at each time point, enabling us to analyze the severity drift for each rater. In these models, the time-specific rater severity parameters are estimated by assuming that they have temporal independence, namely that $\beta _{rt} \sim i.i.d$. ∀*r*,*t* and *β*_*irt*_ $\sim i.i.d$. ∀*i*,*r*,*t*. In practice, however, the severities between adjacent time points tend to depend on each other, as described in “Introduction. When rater severity is assumed to have a time dependency, we can expect that considering the dependency will be helpful in improving the estimation accuracy of the time-specific severity parameters. For this reason, our study aims to develop a Bayesian extension of the MFRM that assumes time dependency for the time-specific rater severity parameters, based on a Markov modeling approach.

## Proposed model

### Settings

As described above, some of the previous studies that have investigated rater severity drift have considered situations where a performance test offers multiple items and the score data for those items are analyzed simultaneously in a single IRT model that considers the effects of raters, items, times, and some interactions among them. However, in this study, to focus on our main aim, which is to accurately investigate rater severity drifts, we consider situations where a test consists of only one item or where IRT models are applied to each item separately. Specifically, we assume that the observed data ***U*** are defined as a collection of *u*_*jrt*_, which indicate a score assigned to the performance of examinee $j\in \mathcal {J}=\{1, 2, \cdots ,J\}$ for an item by rater $r\in \mathcal {R} = \{1, 2, \cdots ,R\}$ at time point $t \in \mathcal {T}=\{1, 2, \cdots ,T\}$. The scores are given by an ordinal category scale $\mathcal {K} = \{1, 2, \cdots , K\}$. Note that, as in previous studies, a *time point* indicates a time period for continuous rating: an hour, a day, or a rating session of some other significant length of time. This means that each rater evaluates multiple examinees at every time point *t*.

In this setting, the conventional MFRMs with time-specific rater severity parameters, namely, Eqs. (2) and (3), can be rewritten in the same form as

4 $$P_{jrtk} = \frac{\exp {\sum}_{m=1}^{k}\left[ D(\theta_{j} - \beta_{rt} - d_{m}) \right]}{{\sum}_{l=1}^{K} \exp {\sum}_{m=1}^{l}\left[D(\theta_{j} - \beta_{rt} - d_{m}) \right]}.$$

This formula is also consistent with the model introduced by Myford and Wolfe (2009). We call this model *the baseline model* in the following, and we will develop the proposed model as an extension of it.

### Model definition

Assuming data ***U***, the proposed model defines the probability for $u_{jrt} = k \in \mathcal {K}$ as

5 $$P_{jrtk} = \frac{\exp {\sum}_{m=1}^{k}\left[ D (\theta_{j} - \beta_{rt} - d_{rm} ) \right]}{{\sum}_{l=1}^{K} \exp {\sum}_{m=1}^{l}\left[D (\theta_{j} - \beta_{rt} - d_{rm} )\right]}$$

6 $$\begin{cases} \theta_{j} \sim N(0, 1) \\ \beta_{r1} \sim N(0, 1) \\ \beta_{rt} \sim N(\beta_{r,t-1} , \sigma_{r}); t>1 \\ \sigma_{r} \sim LN(\mu_{\sigma}, 1)\\ d_{rm} \sim N(0, 1) \end{cases},$$

where *d*_*rm*_ is a rater-specific step parameter denoting the severity for rater *r* of transitioning from score *m* − 1 to *m*, which is often used to examine the central tendency and the range restriction of each rater (Eckes, 2015; Myford & Wolfe, 2004; Qiu et al., 2022; Uto, 2021a). Moreover, *N*(*μ*,*σ*) indicates a normal distribution of mean *μ* and standard deviation *σ*, and *LN*(*μ*,*σ*) indicates a log-normal distribution of mean *μ* and standard deviation *σ* on the log scale. Moreover, *σ*_*r*_ is a rater-specific standard deviation parameter that reflects the degree of severity drift for rater *r*, and *μ*_*σ*_ is a hand-tuning hyperparameter. The details of *σ*_*r*_ and *μ*_*σ*_ are discussed in “Rater-specific standard deviation parameters” and “Prior distribution on rater-specific standard deviation parameters” sections. For model identification, *d*_*r*1_ = 0 and ${\sum }_{m=2}^{K} d_{rm} = 0$ are assumed.

Comparing Eqs. (4) and (5) shows that the proposed model is consistent with the baseline model when the rater-specific step parameter *d*_*rm*_ is replaced with the rater-independent step parameter *d*_*m*_. The main difference between the two models is the addition of the prior distributions for the model parameters that are defined in Eq. (6). Consequently, the proposed model can be regarded as a Bayesian extension of the baseline model. The use of the rater-specific step parameter *d*_*rm*_ to capture rater effects more flexibly is a notable feature of the proposed model, but this modification is not the main focus of this study.

Next, we will look at the unique features of the proposed model in greater detail.

### Markov modeling for time-specific severity parameters

**Fig. 1:**
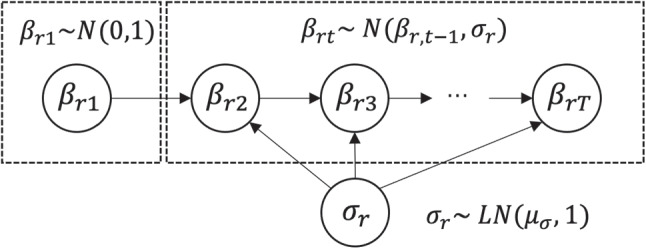
Outline of Markov modeling for time-specific severity parameters

The main feature of the proposed model is that the time-specific rater severity parameters *β*_*rt*_ are modeled as a Markov chain in which the severity at a given time point depends on that at the previous time point. Figure 1 depicts an outline of the formulation for *β*_*rt*_ in the proposed model. As shown by this figure and the model definition, our model assumes that the parameter *β*_*rt*_ (*t* > 1) follows a normal distribution that has the severity at the previous time point *β*_*r*,*t*− 1_ as its mean and the rater-specific standard deviation *σ*_*r*_. This formulation is based on a typical first-order Markov model. Using this, our model can estimate the severity at each time point *β*_*rt*_ while considering its dependency on severity at the previous time point *β*_*r*,*t*− 1_.

### Rater-specific standard deviation parameters

As described above, our model estimates *β*_*rt*_ using *β*_*r*,*t*− 1_ and *σ*_*r*_. Here, *σ*_*r*_ is the rater-specific standard deviation parameter that reflects the degree of severity drift for rater *r*. The proposed model produces small positive values of *σ*_*r*_ for raters whose severity is stable across time because *N*(*β*_*r*,*t*− 1_,*σ*_*r*_) provides high probabilities only around *β*_*r*,*t*− 1_ when *σ*_*r*_ is close to zero. As a result, the adjacent severities *β*_*r*,*t*− 1_ and *β*_*rt*_ tend to have similar values. On the other hand, the proposed model produces large values of *σ*_*r*_ for raters with a stronger severity drift. This makes *N*(*β*_*r*,*t*− 1_,*σ*_*r*_) wider and allows the model to easily produce a value of *β*_*rt*_ that is very different from the value of *β*_*r*,*t*− 1_.

Thus, we can determine the degree of severity drift for each rater from the rater-specific standard deviation parameter estimates.

### Prior distribution on rater-specific standard deviation parameters

Another feature of the proposed model is the addition of a prior distribution on *σ*_*r*_. Specifically, we use a log-normal distribution *LN*(*μ*_*σ*_,1) as the prior distribution, where *μ*_*σ*_ is a hand-tuning hyperparameter. This prior distribution can reflect our assumption of the extent to which rater severity drift occurs across target raters.

**Fig. 2:**
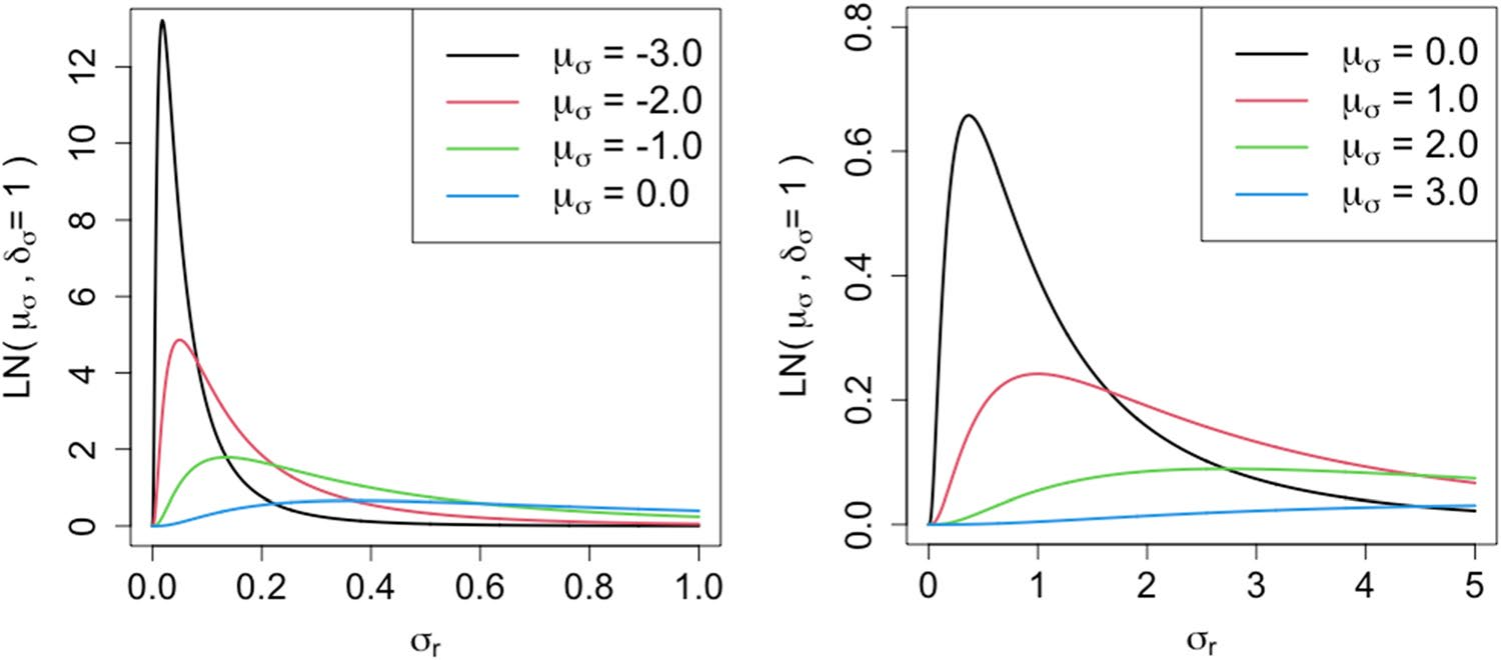
Probability density function for *LN*(*μ*_*σ*_,1) with different values of *μ*_*σ*_. The figure on the *left* depicts the functions with *μ*_*σ*_ ≤ 0.0, and the figure on the *right* depicts those with *μ*_*σ*_ ≥ 0.0

Figure 2 depicts the probability density functions for log-normal distributions with various mean values. If we have a strong prior knowledge that no, or only a few, raters have strong severity drift, then we can reflect this knowledge by selecting a small value for *μ*_*σ*_. As *μ*_*σ*_ decreases, the prior distribution tends to provide small positive values for *σ*_*r*_ overall, as shown in Fig. 2. Because a smaller *σ*_*r*_ indicates a weak rater severity drift, we can see that setting a small value of *μ*_*σ*_ reflects the assumption that no, or only a few, raters have strong severity drift. Conversely, if we assume that there are likely raters with strong severity drifts, then selecting a larger value for *μ*_*σ*_ will ensure that the prior distribution can easily provide large values for *σ*_*r*_.

We recommend using *μ*_*σ*_ with less than − 2 when we have a strong assumption that no, or only a few, raters have severity drift. This is because *LN*(*μ*_*σ*_,1) for these values of *μ*_*σ*_ becomes a strongly skewed distribution providing high probabilities only for extremely small *σ*_*r*_ values, as shown in Fig. 2. Conversely, we recommend using *μ*_*σ*_ within the range from − 1 to 0 when we assume the existence of various raters with strong severity drifts because *LN*(*μ*_*σ*_,1) for those values of *μ*_*σ*_ allows us to easily produce relatively large values for *σ*_*r*_, as shown in Fig. 2. Note that we discourage using *μ*_*σ*_ > 0 because *LN*(*μ*_*σ*_,1) in this case provides high probabilities for *σ*_*r*_ values that are too large, as shown in the right-side of Fig. 2. We can say, however, that *σ*_*r*_ = 1.0 would be large enough, but *σ*_*r*_ greater than 1.0 is generally too large because the scale of *β*_*rt*_ is consistent with that of *β*_*r*1_, which follows the standard normal distribution.

**Fig. 3:**
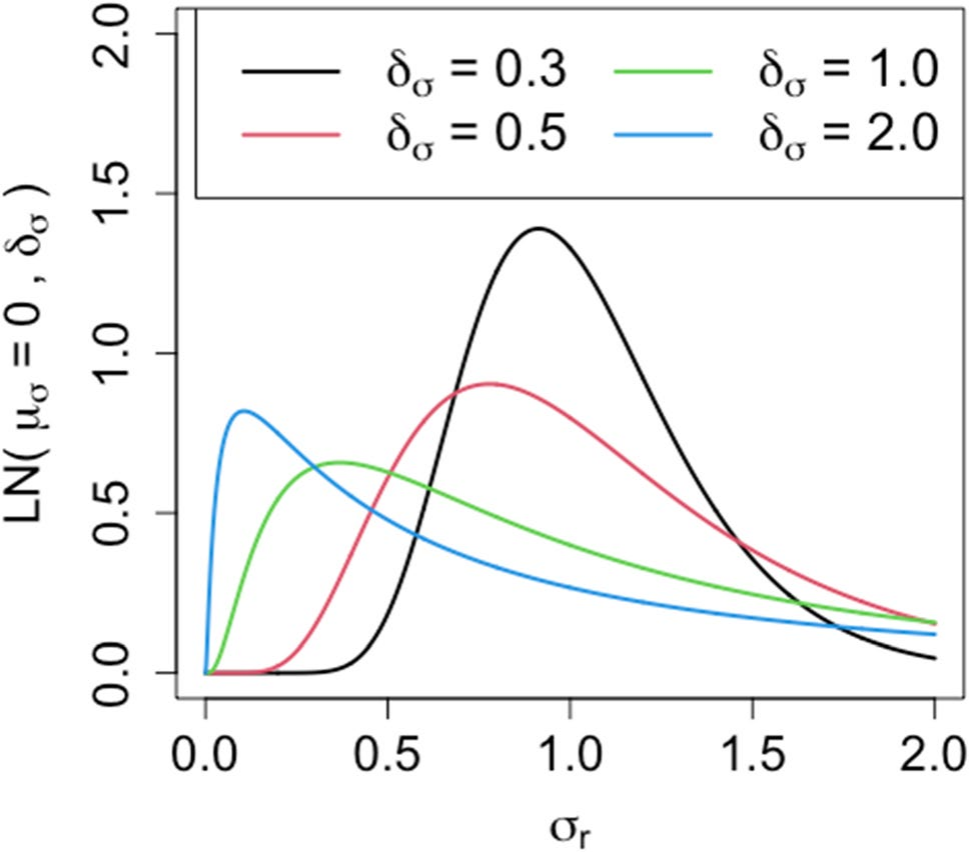
Probability density function for *LN*(0,*δ*_*σ*_) with different values of *δ*_*σ*_

When no prior knowledge is available, the hyperparameter can be selected through model comparison experiments. We will demonstrate this in “Model comparison using information criteria” section. For the remainder of this paper, we use *μ*_*σ*_ = − 2 as the default setting when considering the results of our model comparison experiments.

Note that in this study we fix the standard deviation of the prior distribution to one (i.e., *LN*(*μ*_*σ*_,1)). Although the standard deviation can also be tuned in the same way as the mean value, doing so makes the change in the shape of the prior distribution complex. As an example, Fig. 3 shows the probability density functions for the log-normal distributions with various standard deviation values. We fix the standard deviation to one to facilitate tuning and interpretation of the hyperparameter.

### Asymptotic property and parameter estimation accuracy

As explained in “Model definition” section, the proposed model can be regarded as a Bayesian extension of the baseline model, in which *d*_*m*_ has been replaced with *d*_*rm*_. The parameter estimates of a Bayesian model are known to approach those of its non-Bayesian counterpart as the amount of data increases. This is because the influence of the prior distribution decreases (Gelman et al., 2013). Thus, the parameter estimates of the proposed model asymptotically converge to those of its non-Bayesian counterpart, the baseline model with *d*_*rm*_.

However, when the amount of data is limited, the proposed model estimates the time-specific severity parameters while strongly considering the influence from the prior distributions, including the Markov modeling of the severity parameters. Consequently, when there is time dependency in rater severity and proper prior distributions are set, the proposed model is expected to provide more accurate estimates of the time-specific severity parameters than the baseline model. Furthermore, an improvement in the estimation accuracy of time-specific rater severity parameters is expected to increase the estimation accuracy of other parameters and improve model fitting.

### Bayesian estimation using Markov chain Monte Carlo

Two parameter estimation methods are commonly used for IRT models: marginal maximum likelihood estimation using an expectation–maximization algorithm and maximum a posteriori estimation using a Newton–Raphson algorithm (Baker & Kim, 2004). However, for complex models such as ours, expected a posteriori (EAP) estimation, a type of Bayesian estimation, is known to provide more robust results (Fox, 2010; Uto & Ueno, 2020).

EAP estimates are calculated as the expected value of the marginal posterior distribution for each parameter. The marginal posterior distribution is derived by marginalizing across every parameter except the target parameter. For complex models, however, it is not generally feasible to derive or calculate the marginal posterior distribution due to there being high-dimensional multiple integrals. MCMC, a random sampling-based estimation method, has been widely used in various fields to address this problem, including in IRT studies (Brooks, Gelman, Jones, & Meng, 2011; Fontanella et al., 2019; Fox, 2010; Uto, 2021b; Uto & Ueno, 2020; van Lier et al., 2018; Zhang, Xie, You, & Huang, 2011).

The Metropolis-Hastings-within-Gibbs sampling method (Patz & Junker, 1999) is a common MCMC algorithm used for IRT models. It is simple and easy to implement but requires a long time to converge to the target distribution (Girolami & Calderhead, 2011; Hoffman & Gelman, 2014). An efficient alternative MCMC algorithm is the NUT sampler (Hoffman & Gelman, 2014), which is a variant of the HMC. It was recently developed along with a software package called “Stan” (Carpenter et al., 2017), which makes implementation of a NUT-based HMC easy. Thus, NUT has recently been widely used to perform parameter estimations for various statistical models, including IRT models (Jiang & Carter, 2019; Luo & Jiao, 2018; Uto, 2021b; Uto & Ueno, 2020).

Therefore, we use a NUT-based MCMC algorithm for parameter estimations in the proposed model. The estimation program was implemented in RStan (Stan Development Team, 2018). The Stan code that we developed is provided in the Appendix. The EAP estimates are calculated as the mean of the parameter samples obtained from 2,000 to 5,000 periods using three independent chains. We set a tuning parameter “adapt_delta” in Stan, which controls the step size during a NUT-based MCMC, to 0.98 to reduce the divergent transitions.

## Simulation experiments

In this section, the effectiveness of the proposed model is evaluated through simulation experiments.

### Parameter recovery experiments

This subsection describes the parameter recovery experiment for the proposed model. The following experiment was carried out for different numbers of examinees *J* ∈{100,200,500}, raters *R* ∈{5,10}, and time points *T* ∈{3,5}.

1. For *J* examinees, *R* raters, and *T* time points, randomly generate true model parameters, except for *σ*_*r*_, from the distributions given in Eq. (6). We generated *σ*_*r*_ from *LN*(− 3,1) for 60% of the raters and from *LN*(− 1,1) for the remaining 40% in order to simulate the scenario where more than half of the raters have stable severity while the others have strong severity drift. The number of score categories *K* was fixed at 5 to match the condition of the actual data (see “Experiments using actual data” section).
2. Given the true parameters, randomly generate score data from the proposed model.
3. Estimate the model parameters from the generated data. Here, we assumed *LN*(− 2,1) to be the prior distribution for *σ*_*r*_, the default setting in this study.
4. Calculate the root mean square errors (RMSEs) and the biases between the estimated and true parameters.
5. Repeat the above procedure 50 times, and calculate the average values of the RMSEs and biases.

**Table 1:** Results of parameter recovery experiments for the proposed model

			RMSE				Bias			
*J*	*R*	*T*	*𝜃*_*j*_	*β*_*rt*_	*d*_*rm*_	*σ*_*r*_	*𝜃*_*j*_	*β*_*rt*_	*d*_*rm*_	*σ*_*r*_
100	5	3	0.373	0.175	0.263	0.226	− 0.002	0.014	0.000	0.016
		5	0.372	0.193	0.271	0.158	− 0.006	− 0.007	0.000	− 0.011
	10	3	0.272	0.163	0.268	0.267	0.006	0.009	0.000	0.023
		5	0.283	0.240	0.290	0.251	0.004	0.017	0.000	0.046
200	5	3	0.363	0.165	0.180	0.217	0.001	0.000	0.000	0.025
		5	0.370	0.160	0.192	0.164	0.000	0.002	0.000	0.015
	10	3	0.271	0.143	0.208	0.220	0.000	0.011	0.000	0.014
		5	0.277	0.209	0.216	0.192	− 0.004	− 0.024	0.000	− 0.005
500	5	3	0.365	0.081	0.121	0.179	0.000	− 0.008	0.000	0.000
		5	0.381	0.160	0.135	0.144	0.001	0.004	0.000	0.021
	10	3	0.270	0.118	0.129	0.272	0.001	− 0.007	0.000	0.040
		5	0.269	0.169	0.128	0.241	0.001	0.015	0.000	0.041
Average			0.322	0.165	0.200	0.211	0.000	0.002	0.000	0.019

A result of 0.000 indicates that the value was less than 0.001

For the results shown in Table 1, the *Average* row indicates the RMSE and bias values after averaging over all experimental settings. Based on the RMSE values that were obtained, we can observe some clear trends. (1) The RMSEs for the ability tend to decrease as the number of raters increases. Similarly, the RMSEs for the rater parameters tend to decrease as the number of examinees increases. These tendencies are caused by the increase in the amount of data per parameter. (2) An increase in the number of time points leads to a decrease in the RMSEs for *σ*_*r*_ because the number of the parameters *β*_*rt*_ that are used to estimate *σ*_*r*_ increases. By contrast, an increase in the number of time points tends to increase the RMSEs for *β*_*rt*_ because the amount of data at each time point decreases.

Moreover, Table 1 shows that the average bias was nearly zero overall, indicating that there was no overestimation or underestimation of the parameters. We also confirmed that the Gelman–Rubin statistic $\hat {R}$ (Gelman et al., 2013; Gelman & Rubin, 1992), a well-known convergence diagnostic index, the effective sample size (ESS), and the number of divergent transitions. Consequently, the $\hat {R}$ values were less than 1.1 in all cases (where the average and maximum $\hat {R}$ were 1.000 and 1.009, respectively), indicating that the MCMC runs converged. Furthermore, the ESS values were 7,637 on average and 786 at minimum. According to Zitzmann and Hecht (2019), the ESS over 400 is large enough, and our ESSs satisfy this criterion. Furthermore, we found 46.1 divergent transitions on average in each parameter estimation run, which corresponds to 0.5 % of the total transition. Although some divergent transitions existed, we can conclude that our MCMC runs converged, and we obtained appropriate posterior draws because we confirmed appropriate $\hat {R}$ statistics and sufficient ESSs.

Based on this, we conclude that the parameter estimation for the proposed model can be appropriately conducted by using the MCMC algorithm.

### Effectiveness of Markov modeling for time-specific severity parameters

This subsection investigates the effectiveness of Markov modeling for the time-specific severity parameters *β*_*rt*_. For this purpose, we compared the parameter recovery accuracy between the proposed model and the model without Markov modeling. Specifically, using the data that were generated in procedure 2 of the experiment just discussed, we tested the proposed model under the assumption that there was an i.i.d standard normal distribution for all of the time-specific severity parameters: namely, $\beta _{rt} \sim N(0,1) \forall r, t$. Then, following experimental procedures 4 and 5 in “Parameter recovery experiments” section, the averaged RMSE and the bias between the true and estimated parameter values were calculated. The true parameters were the same as those used in “Parameter recovery experiments” section.

**Table 2:** Results of the parameter recovery experiments for the proposed model without Markov modeling

			RMSE			Bias		
*J*	*R*	*T*	*𝜃*_*j*_	*β*_*rt*_	*d*_*rm*_	*𝜃*_*j*_	*β*_*rt*_	*d*_*rm*_
100	5	3	0.377	0.216	0.269	− 0.008	− 0.010	0.000
		5	0.393	0.306	0.302	− 0.005	− 0.008	0.000
	10	3	0.285	0.253	0.299	0.016	0.025	0.000
		5	0.303	0.491	0.311	− 0.010	− 0.015	0.000
200	5	3	0.374	0.177	0.214	0.003	0.001	0.000
		5	0.377	0.277	0.200	0.001	0.033	0.000
	10	3	0.271	0.169	0.219	0.001	0.002	0.000
		5	0.280	0.247	0.232	− 0.009	− 0.020	0.000
500	5	3	0.376	0.112	0.149	0.001	0.007	0.000
		5	0.373	0.291	0.146	0.000	0.025	0.000
	10	3	0.267	0.169	0.135	0.002	− 0.007	0.000
		5	0.276	0.218	0.158	− 0.002	− 0.014	0.000
*Average*			0.329	0.244	0.219	-0.001	0.002	0.000
*p*-value			0.007	0.001	< 0.001			
*Power*			0.122	0.935	0.266			

A result of 0.000 indicates that the value was less than 0.001

Table 2 shows the results. Note that the results for the rater-specific standard deviation parameter are not reported in it because the model without Markov modeling does not have this parameter. In this experiment, the $\hat {R}$ statistics for all the parameters were less than 1.1 (1.000 on average and 1.003 at maximum), and the ESS values were over 400 (8629 on average and 1492 at minimum). Furthermore, no divergent transitions were observed. These results suggest that the MCMC runs converged and that appropriate posterior draws were obtained.

**Fig. 4:**
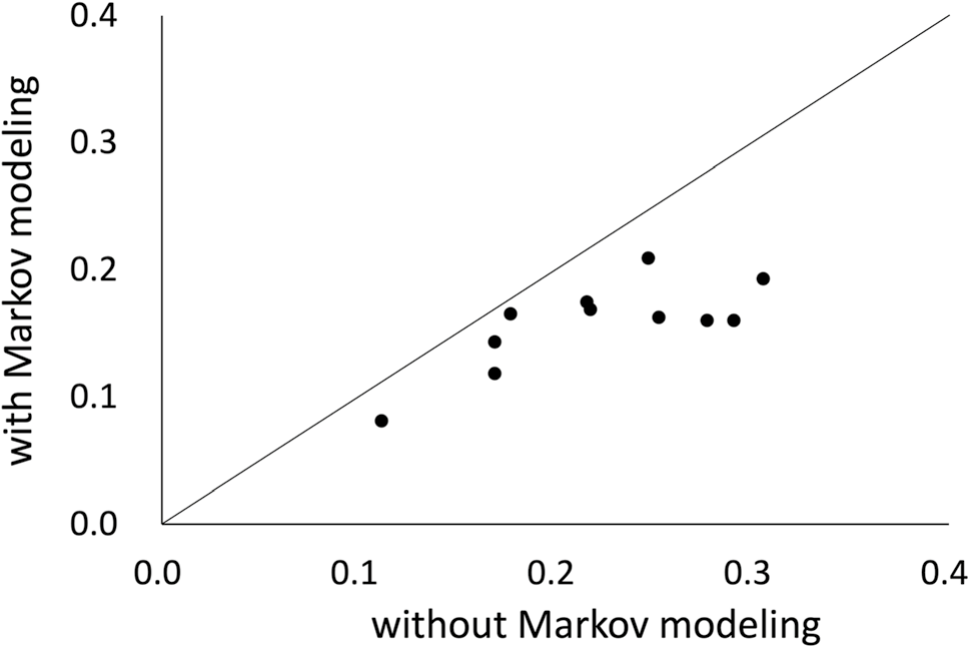
RMSEs for *β*_*rt*_ in the proposed model with and without Markov modeling

Comparing Tables 1 and 2, we can confirm that the incorporation of Markov modeling tends to improve the parameter estimation accuracy overall. The accuracy for *β*_*rt*_ in particular is substantially improved. Figure 4 plots the RMSE values for *β*_*rt*_ in the proposed model with and without Markov modeling. The vertical axis indicates the RMSE values for *β*_*rt*_ in the proposed model, while the horizontal axis indicates the same but without the Markov modeling. Each plot indicates the result for an experimental setting. As this figure shows, the incorporation of Markov modeling improves the RMSEs for *β*_*rt*_ in all cases.

Furthermore, to confirm that the improvements are statistically significant, we conducted a paired t-test for the averaged RMSE values between the proposed model and the model without Markov modeling. We also performed a power analysis with a significance level of 0.05 for the paired t-tests. The *p-value* and *Power* rows in Table 2 show the results, which indicate that the proposed model significantly improves the RMSE for *β*_*rt*_ at a 5% significance level and with a statistical power over 0.80, a threshold that Cohen (1992) recommended. Furthermore, the improvement leads to significant increases in the estimation accuracy of the other parameters at a 5% level, although the statistical powers for them are relatively low.

From these results, we can conclude that using Markov modeling for *β*_*rt*_, which is the main feature of the proposed model, is effective for improving the accuracy of the parameter estimation.

### Evaluation under realistic settings

In the experiments described above, the score data were generated under the assumption of a fully crossed design, where all raters evaluate all examinees. However, in practical situations, the fully crossed design is often infeasible when the number of examinees is large. Thus, to decrease the raters’ assessment workload, the grading of each examinee is performed by a few different raters, who are selected from a collection of raters. In such cases, the amount of data per parameter decreases because of the increased data sparsity. As discussed in “Asymptotic property and parameter estimation accuracy” section, the effectiveness of the proposed model is expected to be emphasized as the amount of data decreases. In this section, we evaluate this point.

**Table 3:** Example of a fully crossed design

	Rater				
	1	2	3	4	5
Examinee 1	$\checkmark$	$\checkmark$	$\checkmark$	$\checkmark$	$\checkmark$
Examinee 2	$\checkmark$	$\checkmark$	$\checkmark$	$\checkmark$	$\checkmark$
Examinee 3	$\checkmark$	$\checkmark$	$\checkmark$	$\checkmark$	$\checkmark$
Examinee 4	$\checkmark$	$\checkmark$	$\checkmark$	$\checkmark$	$\checkmark$
Examinee 5	$\checkmark$	$\checkmark$	$\checkmark$	$\checkmark$	$\checkmark$
Examinee 6	$\checkmark$	$\checkmark$	$\checkmark$	$\checkmark$	$\checkmark$

**Table 4:** Example of a systematic link design

	Rater				
	1	2	3	4	5
Examinee 1	$\checkmark$	$\checkmark$			
Examinee 2		$\checkmark$	$\checkmark$		
Examinee 3			$\checkmark$	$\checkmark$	
Examinee 4				$\checkmark$	$\checkmark$
Examinee 5	$\checkmark$				$\checkmark$
Examinee 6	$\checkmark$	$\checkmark$			

For this purpose, we conducted the same experiment as described in “Parameter recovery experiments” and “Effectiveness of Markov modeling for time-specific severity parameters” section, assuming a more realistic setting where a few raters are assigned to each examinee. Specifically, in experimental procedure 2 in “Parameter recovery experiments” section, we assigned two or three raters to each examinee based on a systematic link design (Shin et al., 2019; Uto, 2021a; Wind and Jones, 2019) and generated score data based on the rater assignment. The systematic link design is a method for creating a rater-examinee assignment under conditions where test linking is possible. Tables 3 and 4 illustrate examples of a fully crossed design and a systematic link design; checkmarks indicate an assigned rater and blank cells indicate that no rater was assigned. The procedures for generating rater-examinee assignment based on the systematic link design are detailed by Uto (2021a). With the exception of the data generation procedure, the procedures for this experiment were the same as those detailed in “Parameter recovery experiments” and “Effectiveness of Markov modeling for time-specific severity parameters” sections. Note that in this section we discuss only the RMSE values because, as can be seen in Tables 1 and 2, the average bias was nearly zero for all cases. As in the simulation experiments above, we confirmed that all MCMC runs in this experiment converged and that sufficient posterior draws were obtained. Specifically, we confirmed for all of the parameters that the $\hat {R}$ statistics were less than 1.1 (1.000 on average and 1.031 at maximum) and that the ESSs were over 400 (10,223 on average and 996 at minimum), although a few divergent transitions existed (33.8 on average, which corresponds to 0.4 % of the total transitions).

**Table 5:** Accuracy of the parameter recovery for the proposed model under systematic link design when two or three raters were assigned to each examinee

			2 raters assigned				3 raters assigned			
*J*	*R*	*T*	*𝜃*_*j*_	*β*_*rt*_	*d*_*rm*_	*σ*_*r*_	*𝜃*_*j*_	*β*_*rt*_	*d*_*rm*_	*σ*_*r*_
100	5	3	0.541	0.254	0.396	0.264	0.455	0.235	0.308	0.272
		5	0.579	0.336	0.402	0.268	0.480	0.315	0.328	0.248
	10	3	0.556	0.391	0.493	0.331	0.472	0.321	0.441	0.302
		5	0.570	0.430	0.504	0.318	0.467	0.362	0.436	0.277
200	5	3	0.535	0.197	0.286	0.294	0.460	0.234	0.263	0.298
		5	0.544	0.205	0.294	0.181	0.467	0.280	0.266	0.219
	10	3	0.557	0.357	0.409	0.375	0.471	0.239	0.329	0.388
		5	0.551	0.339	0.399	0.301	0.478	0.346	0.354	0.262
500	5	3	0.548	0.137	0.199	0.254	0.452	0.101	0.157	0.170
		5	0.550	0.180	0.199	0.170	0.464	0.131	0.160	0.156
	10	3	0.538	0.210	0.280	0.288	0.466	0.202	0.232	0.291
		5	0.554	0.285	0.290	0.227	0.469	0.196	0.247	0.182
Average			0.552	0.277	0.346	0.273	0.467	0.247	0.293	0.255

**Table 6:** Accuracy of the parameter recovery for the proposed model without Markov modeling and under systematic link design when two or three raters were assigned to each examinee

			2 raters assigned			3 raters assigned		
*J*	*R*	*T*	*𝜃*_*j*_	*β*_*rt*_	*d*_*rm*_	*𝜃*_*j*_	*β*_*rt*_	*d*_*rm*_
100	5	3	0.560	0.408	0.380	0.472	0.284	0.344
		5	0.573	0.474	0.404	0.482	0.429	0.384
	10	3	0.580	0.534	0.527	0.497	0.465	0.445
		5	0.625	0.626	0.542	0.523	0.559	0.514
200	5	3	0.555	0.274	0.316	0.469	0.268	0.272
		5	0.573	0.383	0.333	0.479	0.287	0.282
	10	3	0.566	0.397	0.431	0.463	0.271	0.335
		5	0.592	0.582	0.463	0.489	0.439	0.406
500	5	3	0.541	0.174	0.211	0.468	0.137	0.182
		5	0.546	0.289	0.224	0.466	0.281	0.185
	10	3	0.551	0.262	0.292	0.470	0.300	0.240
		5	0.563	0.413	0.313	0.476	0.314	0.269
*Average*			0.569	0.401	0.370	0.480	0.336	0.322
*p*-value			0.005	< 0.001	0.001	0.010	< 0.001	0.001
Power			0.869	0.934	0.189	0.895	0.866	0.251

Table 5 shows the RMSE values for the proposed model under a systematic link design where two or three raters were assigned to each examinee. Furthermore, Table 6 shows the results for the proposed model without Markov modeling, where the *p-value* and *Power* rows indicate the results of the paired t-test and the corresponding power analysis for the averaged RMSE between the proposed model with and the model without Markov modeling.

First, according to these tables and Tables 1 and 2, the parameter estimation accuracy tends to decrease as the number of raters assigned to each examinee decreases. This is caused by a decrease in the amount of data per parameter, which is a reasonable tendency. Next, comparing Tables 5 and 6, the proposed model with Markov modeling tends to have lower RMSE values, especially for the rater parameters *β*_*rt*_ and *d*_*rm*_. It also improves the average RMSE values for all of the parameters. Furthermore, the improvements in *β*_*rt*_ are statistically significant at a 5% significance level and with a statistical power over 0.80.

Next, we take a look at the averaged improvement in the RMSE of *β*_*rt*_ by incorporating Markov modeling. According to Tables 1, 2, 5, and 6, the improvement in the average RMSE for *β*_*rt*_ is 0.079 under the fully crossed design, 0.089 under the systematic design with three assigned raters, and 0.124 under the systematic design with two assigned raters. This result suggests that the effectiveness of the proposed model tends to increase as the amount of data per parameter decreases.

### Influence of the prior distribution on rater-specific standard deviations

The proposed model assumes a prior distribution on the rater-specific standard deviation parameter *σ*_*r*_. As previously explained, this prior distribution reflects our assumption regarding the extent to which rater severity drift occurs across target raters, and the distribution can be controlled by the hyperparameter *μ*_*σ*_. In this subsection, we investigate how the prior distribution influences *β*_*rt*_ estimates.

To do this, we conducted the following experiment.

1. For *J* = 500 examinees, *R* = 10 raters, and *T* = 5 time points, true model parameters were randomly generated from the distributions following procedure 1 in “Parameter recovery experiments” section.
2. Given the true parameters, score data was randomly generated from the proposed model following procedure 2 in “Parameter recovery experiments” section. We generated three datasets in which the fully crossed design and the systematic link design with two or three assigned raters were applied, respectively.
3. The model parameters were estimated from each dataset by using the proposed model with three different hyperparameters *μ*_*σ*_ ∈{− 5,− 2,0} and the proposed model without Markov modeling (i.e., $\beta _{rt} \sim N(0, 1); \forall r, t$).

**Fig. 5:**
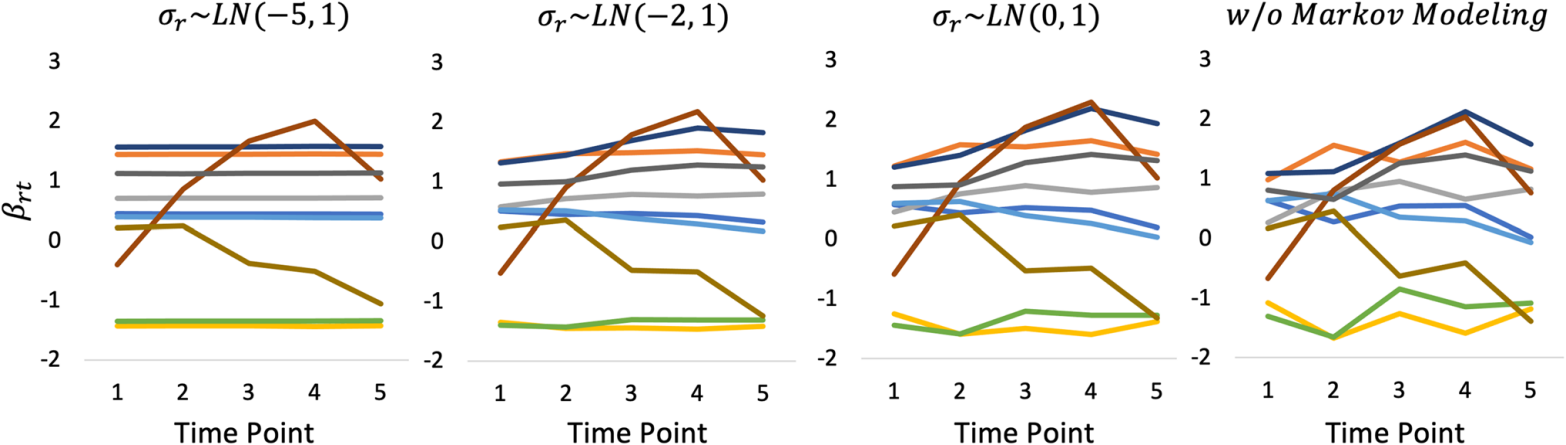
*β*_*rt*_ estimates under a systematic link design when two raters were assigned

**Fig. 6:**
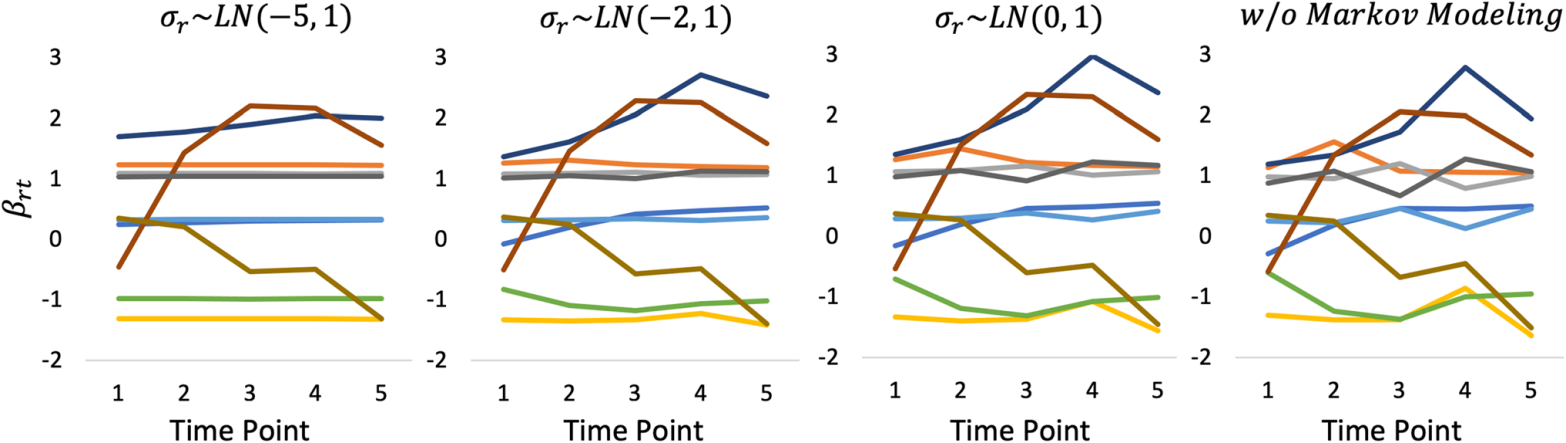
*β*_*rt*_ estimates under a systematic link design when three raters were assigned

**Fig. 7:**
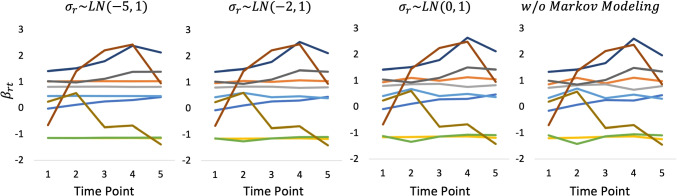
*β*_*rt*_ estimates under a fully crossed design

**Fig. 8:**
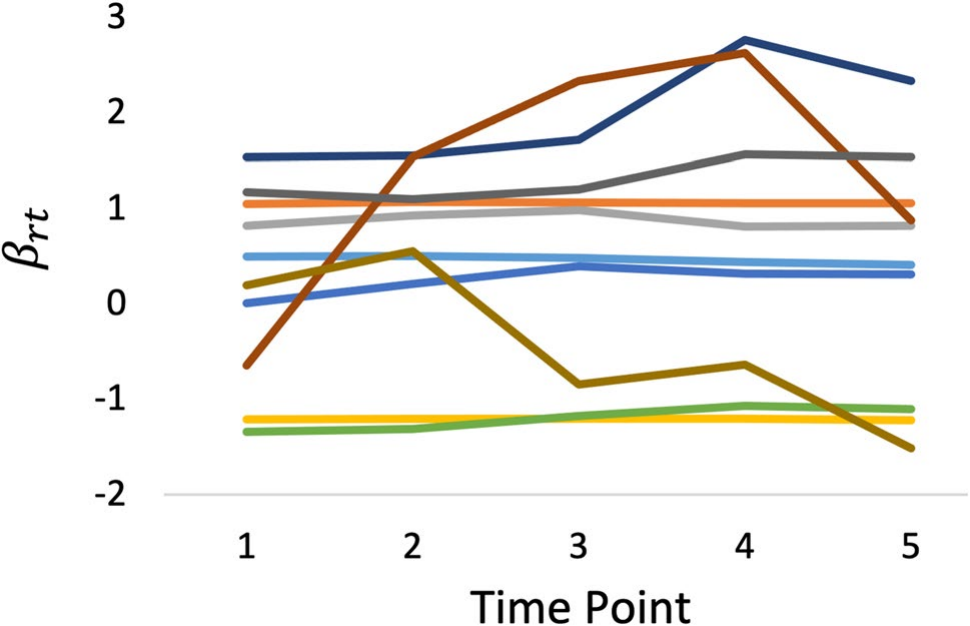
True *β*_*rt*_ values corresponding to Figs. 5, 6, and 7

Figures 5, 6, and 7 show the estimated *β*_*rt*_ that was obtained using the three datasets, respectively. Moreover, Fig. 8 shows the true values of *β*_*rt*_ that were generated in experimental procedure 1. In each figure, the horizontal axis indicates the time point, the vertical axis indicates the true or estimated *β*_*rt*_ values, and each line indicates a rater.

A comparison of these figures shows that in all models the estimated *β*_*rt*_ values approach the true values as the number of raters per examinee is increased. This shows that the influence of the prior distributions, including Markov modeling for *β*_*rt*_, decreases in the proposed model as the amount of data increases, which supports our discussion in “Asymptotic property and parameter estimation accuracy” section.

Conversely, the influence of the prior distributions and Markov modeling increases when the amount of data per parameter decreases, as in the systematic link designs. For example, Figs. 5 and 6 show that when we use a strongly skewed prior distribution *LN*(− 5,1) by selecting *μ*_*σ*_ = − 5, the proposed model tends to estimate the time-specific severity parameters *β*_*rt*_ in such a way that their temporal changes become smaller overall. In contrast, when we assume a weakly informative (flatter) prior distribution *LN*(0,1) for the proposed model or a time-independent *β*_*rt*_ for the proposed model without Markov modeling, the estimated *β*_*rt*_ become unstable over time, even for raters whose true *β*_*rt*_ values are stable. Using the proposed model with a moderate setting for the prior distribution *LN*(− 2,1), namely *μ*_*σ*_ = − 2, provides relatively good estimates for *β*_*rt*_ overall.

From these results, we can confirm that the prior distribution *LN*(*μ*_*σ*_,1) works well, as we expected in “Prior distribution on rater-specific standard deviation parameters” sections. Note that, as was explained in “Prior distribution on rater-specific standard deviation parameters” section, the hyperparameter can be selected either by practitioners when they have strong prior knowledge or by a model selection approach when no prior knowledge exists. An example of using information criteria for hyperparameter selection is described in “Model comparison using information criteria” section.

## Experiments using actual data

In this section, we evaluate the effectiveness of the proposed model through experiments using actual data.

### Actual data

For this experiment, we collected actual data from an essay writing test as follows:

1. We recruited 134 Japanese university students as participants. The participants were asked to complete an essay-writing task. This was created by translating a task used in the National Assessment of Educational Progress (NAEP) assessments (Persky, Daane, & Jin, 2003) into Japanese. No specific or preliminary knowledge was needed to complete the task.
2. The written essays were evaluated by ten raters using a rubric with five score categories, which was created by translating a rubric used in the NAEP assessments. Each rater was asked to complete their evaluation of the 134 essays in four days while grading 1/4 of them each day. The order of the given essays was randomized for each rater. In this experiment, we regard *a day* as *a time point*.
3. We also collected score data from intentionally biased raters. Specifically, we gathered the other five raters and asked them to grade essays according to the following instructions.
  **Rater 11:** Grade essays while gradually increasing severity so that the average scores decrease day by day.
  **Rater 12** Grade essays while gradually decreasing severity so that the average scores increase day by day.
  **Rater 13:** Grade essays while changing severity each day so that average scores change every day. Specifically, increase the severity on the second day compared to that on the first day, decrease the severity on the third day compared to that on the first day, and increase the severity on the fourth day compared to that on the second day.
  **Rater 14:** Grade essays mainly using score categories 2, 3, and 4.
  **Rater 15:** Grade essays mainly using score categories 1, 3, and 5.
  The instructions for the first three raters were intended to imitate strong rater drift. Those for the last two raters were given so that we could investigate the influence of the rater-specific step parameter *d*_*rm*_. Although, as was mentioned in “Model definition” section, the modification of *d*_*m*_ to *d*_*rm*_ is not central to the proposed model. We refer to these five raters as *control raters* for the remainder of this paper.

### Model comparison using information criteria

In this section, we describe model comparison experiments using the actual data. In various research domains, model comparisons are typically conducted using information criteria, such as the Akaike information criterion (AIC) (Akaike, 1974), the Bayesian information criterion (BIC) (Schwarz, 1978), the widely applicable information criterion (WAIC) (Watanabe, 2010), and the widely applicable Bayesian information criterion (WBIC) (Watanabe, 2013). The AIC and BIC are applicable when maximum likelihood estimation is used to estimate model parameters, whereas the WAIC and the WBIC are applicable with Bayesian estimation using MCMC or variational inference methods. With the recent increase in complex statistical and machine learning models, various studies have used the WAIC and the WBIC with a Bayesian estimation (Almond, 2014; Luo & Al-Harbi, 2017; Vehtari, Gelman, & Gabry, 2017). Because this study uses a Bayesian estimation based on MCMC, we use the WAIC and WBIC. The model that minimizes these criteria is regarded as optimal.

**Table 7:** Model comparison of the proposed model with different hyperparameters

	Full Data		w/o Control Raters	
	WAIC	WBIC	WAIC	WBIC
*μ*_*σ*_ = − 3	4,665.52	2,828.06	3,138.25	1,907.14
*μ*_*σ*_ = − 2	**4,662.44**	**2,821.80**	**3,133.86**	**1,903.95**
*μ*_*σ*_ = − 1	4,663.85	2,823.12	3,134.41	1,914.35
*μ*_*σ*_ = 0	4,667.99	2,833.57	3,138.52	1,924.21

Bold texts indicate the minimum values for each condition

We first conducted a model comparison experiment to determine the hyperparameter *μ*_*σ*_. The task of determining optimal hyperparameters is generally known as hyperparameter optimization, which can be seen as a subtask of model selection (Bertrand et al., 2022; Feurer & Hutter, 2019; Watanabe, 2010; 2013). Typical hyperparameter optimization approaches are empirical Bayes and cross-validation (Bertrand et al., 2022; Feurer & Hutter, 2019; McInerney, 2017; Pedregosa, 2016; Watanabe, 2010; 2013). Empirical Bayes determines hyperparameters based on the marginal likelihood. However, because the exact calculation of the marginal likelihood is generally infeasible, we usually use BIC and WBIC, which are approximations of the marginal likelihood (Watanabe, 2013). Furthermore, AIC and WAIC often substitute cross-validation because (1) cross-validation generally requires a significantly higher computational cost than WAIC and (2) AIC and WAIC are approximations of the generalization error, as with cross-validation (Pedregosa, 2016; Watanabe, 2010). For these reasons and those discussed in the previous paragraph, we used the two information criteria WAIC and WBIC for determining the hyperparameter *μ*_*σ*_. Specifically, we calculated the WAIC and WBIC for the proposed model by using the data with and without the control raters, respectively, while changing the hyperparameter value *μ*_*σ*_ ∈{− 3,− 2,− 1,0}. Table 7 shows the results of these calculations, with the minimum values for each condition being given in bold. The *Full Data* column shows the results for the dataset consisting of the ten normal raters and the five control raters, and the *w/o Control Rater* column shows the results for the dataset consisting of only the ten normal raters. The table indicates that the WAIC and WBIC are minimized when *μ*_*σ*_ = − 2 for both datasets, suggesting that *μ*_*σ*_ = − 2 is optimal. Thus, we used *μ*_*σ*_ = − 2 for the remaining experiments.

Next, we compared the proposed model with the baseline model defined in Eq. (4). In this experiment, we calculated the WAIC and the WBIC for both the proposed model and the baseline model, with and without the Markov modeling for *β*_*rt*_, and using the two datasets. We estimated the baseline model by using the MCMC, just as the proposed model did. The prior distributions were also consistent with the proposed model. To be more specific, we assumed *𝜃*_*j*_, *β*_*rt*_, and $d_{m} \sim N(0, 1)$ for the original baseline model and *𝜃*_*j*_, *β*_*r*1_, $d_{m} \sim N(0, 1)$, $\beta _{rt(t>1)} \sim N(\beta _{r,t-1} , \sigma _{r})$, and $\sigma _{r} \sim LN(\mu _{\sigma }=-2, 1)$ for the baseline model with Markov modeling. Note that the step parameter is the only difference between the proposed model and the baseline model with Markov modeling. Similarly, the step parameter is the only difference between the baseline model and the proposed model without Markov modeling. Thus, by comparing the performance of these pairs, we can determine the effectiveness of changing the step parameter *d*_*m*_ to the rater-specific one *d*_*rm*_.

**Table 8:** Model comparison of the proposed model and the baseline model

	Full Data		w/o Control Raters	
	WAIC	WBIC	WAIC	WBIC
Proposed model	**4,662.44**	**2,821.80**	**3,133.86**	1,903.95
w/o Markov modeling	4,686.79	2,879.36	3,152.27	1,961.55
Baseline model	4,951.87	2,908.21	3,279.42	1,956.79
with Markov modeling	4,924.02	2,843.70	3,257.30	**1,896.22**

Bold texts indicate the minimum values for each condition

Table 8 shows the results of this comparison, with the minimum values for each setting being given in bold. The results show that the criteria values for the proposed model deteriorate when Markov modeling is omitted in all cases. Furthermore, the criteria values for the baseline model improved when Markov modeling was added. These results demonstrate how effective using Markov modeling for *β*_*rt*_ is in improving the model fitting.

By comparing the baseline model with the proposed model without Markov modeling, we can see that the proposed model provided the better criteria values in almost all cases, the exception being the case using the WBIC in the dataset of the ten normal raters. Furthermore, a comparison between the proposed model and the baseline model with Markov modeling shows the same results. These results suggest that the use of the rater-specific step parameters *d*_*rm*_ is likely to be effective.

Note that, as in the simulation experiments, we confirmed that all the MCMC runs in the above experiments were converged appropriately and provided posterior draws with enough ESSs, although a few divergent transitions existed. Specifically, the average and maximum $\hat {R}$ statistics were 1.000 and 1.009, respectively, which are less than 1.1. Furthermore, the average and minimum ESSs were 13,714 and 508, respectively, which are over 400. The average number of divergent transitions was 21.1.

### Interpretation of the rater parameters

**Fig. 9:**
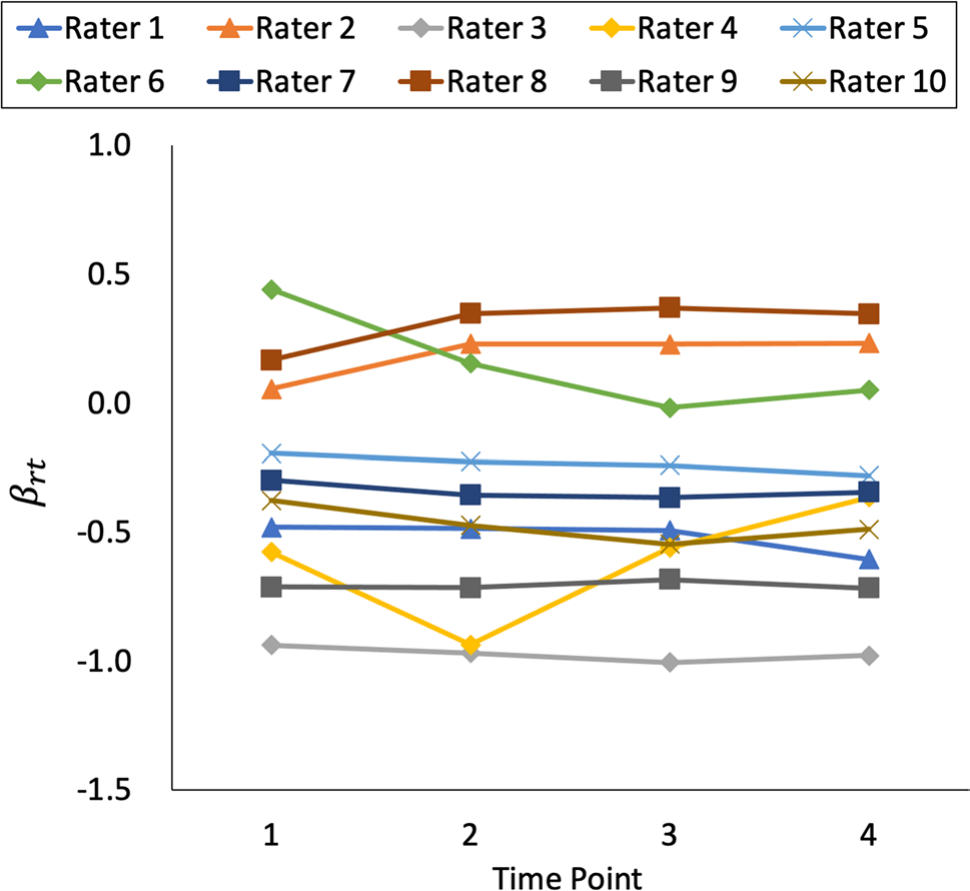
Estimates of *β*_*rt*_ for the ten normal raters

**Fig. 10:**
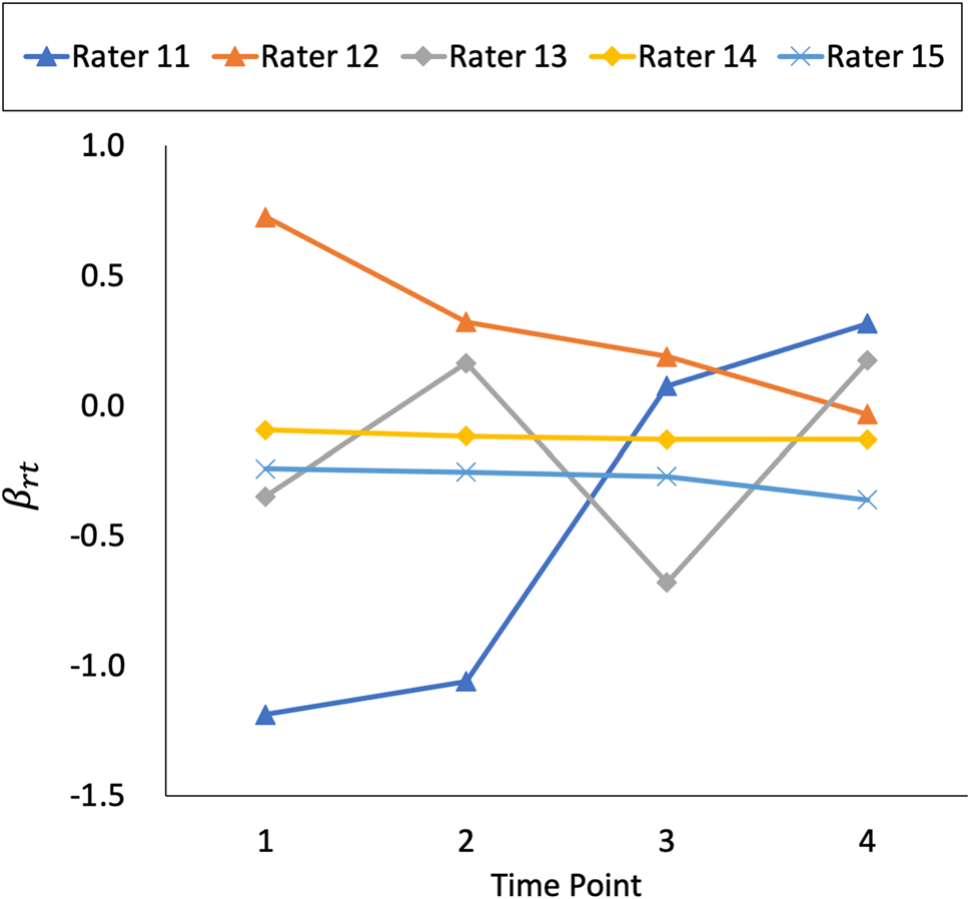
Estimates of *β*_*rt*_ for the five control raters

In this subsection, we provide an interpretation of the rater parameters. Table 9 shows the rater parameter estimates of the proposed model for the full data. In it, the first ten raters are the normal raters and the latter five raters are the control raters. Figures 9 and 10 show the estimates of *β*_*rt*_ for the ten normal raters and the five control raters, respectively.

**Table 9:** Parameter estimates based on the proposed model

*r*	*β*_*r*1_	*β*_*r*2_	*β*_*r*3_	*β*_*r*4_	*d*_*r*2_	*d*_*r*3_	*d*_*r*4_	*d*_*r*5_	*σ*_*r*_
1	− 0.48	− 0.48	− 0.49	− 0.60	− 2.06	− 0.18	0.62	1.63	0.13
2	0.05	0.23	0.23	0.23	− 0.86	− 0.52	0.55	0.83	0.16
3	− 0.94	− 0.97	− 1.00	− 0.98	− 0.80	− 1.54	0.29	2.05	0.11
4	− 0.58	− 0.94	− 0.56	− 0.36	− 1.70	− 1.21	0.78	2.12	0.37
5	− 0.19	− 0.23	− 0.24	− 0.28	− 2.39	− 0.30	0.83	1.86	0.11
6	0.44	0.15	− 0.02	0.05	− 1.57	− 0.61	0.46	1.73	0.26
7	− 0.30	− 0.36	− 0.36	− 0.34	− 1.08	− 0.11	0.61	0.58	0.10
8	0.17	0.35	0.37	0.35	− 1.54	− 0.75	0.85	1.43	0.17
9	− 0.71	− 0.71	− 0.68	− 0.72	− 0.99	− 0.38	0.44	0.92	0.10
10	− 0.38	− 0.47	− 0.55	− 0.49	− 1.51	− 0.13	0.39	1.25	0.15
11	− 1.19	− 1.06	0.08	0.32	− 1.82	− 0.78	1.03	1.57	0.67
12	0.73	0.32	0.19	− 0.03	− 1.01	− 0.09	0.20	0.90	0.33
13	− 0.35	0.16	− 0.68	0.17	− 1.17	− 0.74	0.76	1.15	0.72
14	− 0.09	− 0.12	− 0.13	− 0.13	− 1.47	− 0.53	0.37	1.63	0.09
15	− 0.24	− 0.25	− 0.27	− 0.36	0.28	− 1.31	1.50	− 0.47	0.12

According to Table 9 and the figures, we can confirm that the tendency for rater severity drift varies across raters. For example, among the normal raters, Rater 6 gradually became lenient during the first three days, whereas Raters 2 and 8 became severe during the first two days. Rater 4 showed a relatively strong rater drift where the severity changed each day. By contrast, the other raters were likely to have either weak severity drift or no severity drift because their severity values were stable over time. Among the control raters, the severity of Rater 11 gradually increased and that of Rater 12 gradually decreased. The severity of Rater 13 fluctuated up and down each day. These tendencies are consistent with the expected outcomes of the instructions that we gave to these raters, meaning that they followed our instructions and that the proposed model succeeded in estimating their behaviors.

From the information presented in Table 9, we can also confirm that the rater-specific standard deviation *σ*_*r*_ appropriately reflects the strength of the rater severity drifts. For example, the proposed model gave large values of *σ*_*r*_ for Raters 4, 11, 12, and 13, all of whom showed strong severity drift. Conversely, it provided low values of *σ*_*r*_ for the raters whose severity was stable.

**Fig. 11:**
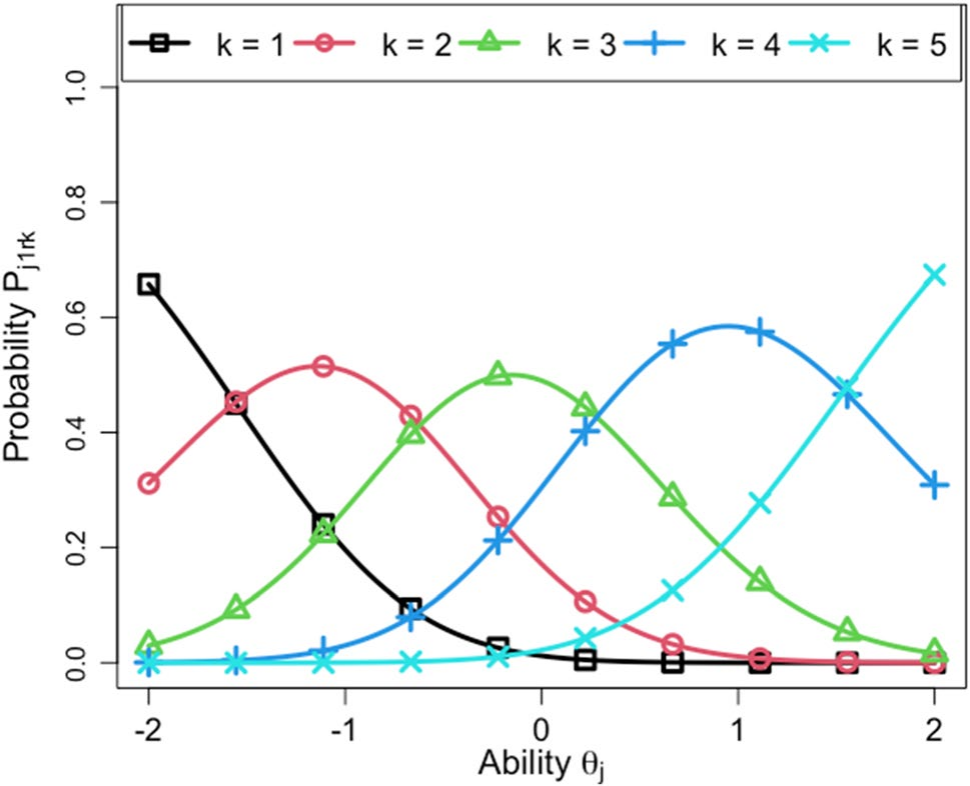
Probability distribution of the proposed model for Rater 14

**Fig. 12:**
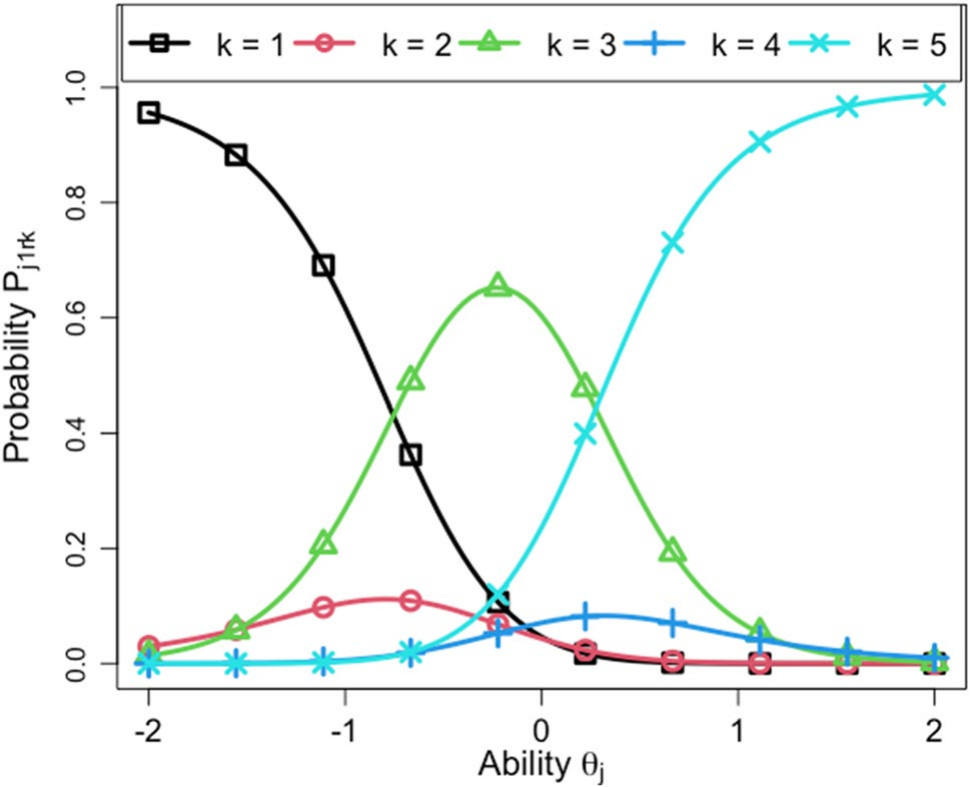
Probability distribution of the proposed model for Rater 15

Table 9 also shows that the step parameters *d*_*rm*_ differed among raters, meaning that they had different criteria for the score categories. To confirm whether the step parameters were estimated as we expected, Figs. 11 and 12 plot the response probability based on the proposed model at time point *t* = 1 for Raters 14 and 15, who were given instructions about the usage of the score categories. In these figures, the horizontal axis shows the examinee ability *𝜃*_*j*_ and the vertical axis shows the probability *P*_*j*(*t*= 1)*rk*_. We can see that Rater 14 tended to overuse the central score categories, namely, scores 2, 3, and 4. Rater 15, on the other hand, tended to prefer score categories 1, 3, and 5, while avoiding scores 2 and 4. These results are consistent with the instructions given to these raters, suggesting that the rater-specific step parameters *d*_*rm*_ can properly capture each rater’s criteria for the score categories.

## Conclusions

In this study, we proposed a Bayesian MFRM that considers a time dependency of the time-specific rater severity parameters to estimate rater severity drift accurately. Specifically, in the proposed model, the time-specific severity parameters for each rater were modeled as a Markov chain such that the severity at a time point depended on that at the previous point. Furthermore, we designed the proposed model so that it has unique components: namely, the rater-specific standard deviation parameters and the prior distribution for them. A NUT variant of the HMC algorithm for the proposed model was implemented using the software package Stan. Using simulation and actual data experiments, we demonstrated the following features: 1) The proposed model can estimate the time-specific rater severity parameters more accurately than conventional models that assume time independence for their parameters. 2) The rater-specific standard deviation parameters provide summarized information representing the degree of severity drift for each rater. 3) The proposed model can represent our prior knowledge of how often rater severity drift occurs as the prior distribution of the rater-specific standard deviation parameters. 4) The parameter estimates of the proposed model approach those of its non-Bayesian counterpart as the amount of data increases. 5) An improvement in the estimation accuracy of the time-specific rater severity parameters leads to an increase in the estimation accuracy of the other parameters, and to an improvement in model fitting.

In future studies, we plan to evaluate the effectiveness of the proposed model using various and more massive datasets. In this study, we assumed a situation where there was only one test item. Going forward, we hope to extend the proposed model to handle situations with multiple test items. We would also like to investigate the effectiveness of using multi-order Markov models for the time-specific rater severity parameters. In this study, we only used the first-order Markov model, so extending it in this fashion would allow us to investigate a longer-term dependency.

## Appendix:

The Stan code for the proposed model is as follows.

Note that we implemented the proposed model based on the second-line form in the following equation:

$$\begin{array}{@{}rcl@{}} P_{jrtk} &=& \frac{\exp {\sum}_{m=1}^{k}\left[ D (\theta_{j} - \beta_{rt} - d_{rm} ) \right]}{{\sum}_{l=1}^{K} \exp {\sum}_{m=1}^{l}\left[D (\theta_{j} - \beta_{rt} - d_{rm} )\right]} \\ &=& \frac{\exp \left[ D \left(k (\theta_{j} - \beta_{rt}) - {\sum}_{m=1}^{k} d_{rm} \right) \right]}{{\sum}_{l=1}^{K} \exp \left[ D \left(l (\theta_{j} - \beta_{rt}) - {\sum}_{m=1}^{l} d_{rm} \right)\right]}. \end{array}$$

The list *c* defined in the *transformed data* block in the Stan code corresponds to the constants *k* and *l* that appear between *D* and *𝜃*_*j*_ in the above equation.
